# Drift Rather than Selection Dominates MHC Class II Allelic Diversity Patterns at the Biogeographical Range Scale in Natterjack Toads *Bufo calamita*

**DOI:** 10.1371/journal.pone.0100176

**Published:** 2014-06-17

**Authors:** Inga Zeisset, Trevor J. C. Beebee

**Affiliations:** 1 School of Pharmacy and Biomolecular Sciences, University of Brighton, Brighton, United Kingdom; 2 School of Life Sciences, University of Sussex, Brighton, United Kingdom; Swansea University, United Kingdom

## Abstract

Study of major histocompatibility complex (MHC) loci has gained great popularity in recent years, partly due to their function in protecting vertebrates from infections. This is of particular interest in amphibians on account of major threats many species face from emergent diseases such as chytridiomycosis. In this study we compare levels of diversity in an expressed MHC class II locus with neutral genetic diversity at microsatellite loci in natterjack toad (*Bufo (Epidalea) calamita*) populations across the whole of the species’ biogeographical range. Variation at both classes of loci was high in the glacial refugium areas (REF) and much lower in postglacial expansion areas (PGE), especially in range edge populations. Although there was clear evidence that the MHC locus was influenced by positive selection in the past, congruence with the neutral markers suggested that historical demographic events were the main force shaping MHC variation in the PGE area. Both neutral and adaptive genetic variation declined with distance from glacial refugia. Nevertheless, there were also some indications from differential isolation by distance and allele abundance patterns that weak effects of selection have been superimposed on the main drift effect in the PGE zone.

## Introduction

The basis of adaptive rather than neutral genetic variation has become increasingly accessible in recent years as loci under selection are identified and characterised. Some of the most popular genes used in this context are those belonging to the major histocompatibility complex (MHC). These genes play an important role in the adaptive immune response of vertebrates. MHC class I molecules present intracellular pathogen peptides to CD8+ T lymphocytes (T cells), primarily in response to viral infections, whereas MHC class II molecules (composed of α and β subunits) present extracellular pathogen peptides to CD4+ T cells after invasion by bacteria and fungi [1]. Although there is some variation among vertebrates in MHC gene structure, MHC class II β genes in the amphibian *Xenopus laevis* are made up of six exons with an exon-intron organization similar to that of a typical mammalian class II β gene. Exon 2 encodes the β - 1 domain which includes most of the antigen binding sites (ABS) of the beta domain and is the most polymorphic region of the gene [2], [3]. Due to high selective pressure on MHC genes, variation tends to be high, particularly at the ABS [4]. These sites encode amino acid residues involved in the recognition and binding of foreign peptides [5].

Frequency dependent selection, where bearers of common alleles are likely to be more susceptible to diseases and specific alleles can confer resistance [6], [7] or heterozygote advantage [8], [9] are believed to be common mechanisms involved in shaping MHC diversity. Low diversity at MHC loci has been implicated in elevating vulnerability to disease [10], [11], though several species have not shown demonstrable ill effects from limited MHC variation [12], [13]. Single MHC class I or II alleles confer resistance to specific diseases in many taxa [14], [15], [16] and some studies suggest that it is the prevalence of parasites which maintain high levels of MHC variation [17], [18], [19]. However, in many cases stochastic events, rather than selection influence MHC variation and variation at neutral markers is often correlated with that of MHC loci, e.g. [11], [20], [21]. The mechanisms driving MHC evolution have therefore not been fully resolved. Natural selection, demographic processes such as drift and gene flow as well as mutation rate are all likely to play a role.

Few studies have investigated MHC variation and neutral genetic variation across entire biographical ranges of species and most of these involve only few populations or species within limited ranges [12], [22], [23], [24], [25], [26]. It is therefore particularly interesting to compare diversity at neutral markers and functionally important genes in species at wide biogeographical scales. In particular, species with large ranges and which have been subjected to population bottlenecks in areas of their distribution are ideal for comparisons of neutral and adaptive genetic variation. Postglacial expansion of amphibians from glacial refugia provides useful examples. Most European species survived the last glaciations, peaking around 20,000 years ago, in southern refugia from which they subsequently colonised northern Europe in the postglacial Holocene period [27]. Furthermore, amphibians are experiencing high rates of global decline [28], [29], mainly due to habitat degradation and loss [30] but also because of emerging infectious diseases such as the chytrid fungus *Batrachochytrium dendrobatidis* (Bd, [31]). This pathogen precipitated the decline of at least two species in parts of Iberia [32], but many other infected species appear largely unaffected [33], [34], [35]. As MHC class II molecules play an important role in mounting acquired immune responses to fungi, and MHC variability is often associated with immunocompetence [36], it is likely that MHC- dependent resistance mechanisms contribute to fighting Bd infections [37]. A recent study on leopard frogs, for example, showed an association between MHC class II genotypes and survival of Bd infections [38]. Information on MHC loci, and MHC class II β 1 genes in particular, is now available for several amphibians including members of the genus *Bombina, Alytes* [39], *Rana* [40], [41], *Bufo* [42], [43], *Espadarana* and *Sachatamia* [44] and *Triturus* [12], [45], as well as for model organisms such as *Xenopus*, *Silurana* and *Ambystoma* [46], [47], [48], [49].

Here we report the results of a study of MHC and microsatellite diversity across the entire biogeographical range of *Bufo calamita*, an amphibian with glacial refugia in Iberia and south-west France, which now also inhabits much of north and central Europe [50]. We recently characterized the entire exon 2 of an expressed MHC class II β locus (locus B, [43]) in *B. calamita* and here we provide evidence that this locus is or has been under selection in this species. We then tested the hypothesis that effects of selection on this locus during postglacial expansion resulted in patterns of diversity different from those of microsatellites, which were presumed to be primarily consequent on genetic drift.

## Materials and Methods

### Sampling Strategy

**Figure 1:**
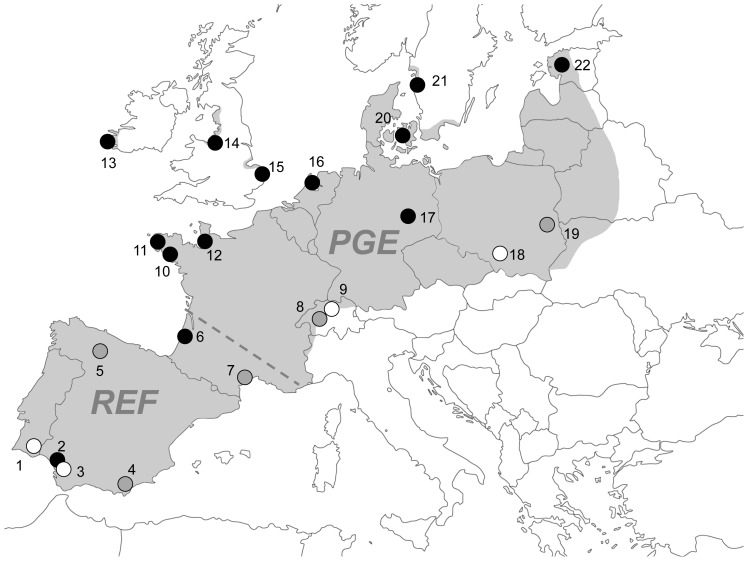
Natterjack toad distribution and sampling sites for microsatellite and MHC analyses. Dark shading indicates natterjack toad distribution in Europe. Black circles indicate sites for which both MHC and microsatellite data was collected, white circles indicate MHC data only and grey circles microsatellite data only. Sampling sites (area or nearest town) were: 1. Algarve, Portugal; 2. Seville, Spain; 3. Doñana, Spain; 4. Almeria, Spain; 5. Leon, Spain; 6. Bordeaux, France; 7. Carmargue, France; 8 and 9 near Zurich, Switzerland; 10. Carnac, France; 11. Penmarch, France; 12. Cherbourg, France; 13. Kerry, Ireland; 14. Birkdale, UK; 15. Winterton, UK; 16. Texel, Netherlands; 17. Halle, Germany; 18. Bukowno, Poland; 19. Bielowieza, Poland; 20. Zealand, Denmark; 21. Uddevalla, Sweden; 22. Parnu, Estonia. The dashed line indicates the approximate division between the glacial refugia (REF) and postglacial expansion (PGE) areas. Map modified from d-maps.com.

For MHC analyses we extracted DNA from 325 individuals from 17 populations of *B. calamita* distributed over the entire species’ range (see Figure 1 and Table 1). Thirteen of those populations were used in previous studies of microsatellite diversity [50], [51] but samples from five further populations used in those studies were no longer available (grey circles in Figure 1). We therefore supplemented the study with samples from four new sites (white circles in Figure 1) to maintain coverage of the full biogeographic range. In all cases free swimming tadpoles (Gosner stages 25–30) were sampled and instantly sacrificed by immersion in ethanol (a method approved by the British Home Office). All UK samples were authorized and licensed by Natural England, the statutory government organisation responsible for wildlife conservation. *Bufo calamita* is a protected species in Britain and in some of the other countries providing samples. In all cases the appropriate permissions were obtained by the samplers in those countries. *Bufo calamita* is a vertebrate but no ethical permissions were required for this study because it only required instant sacrifice of larvae, which does not come under ethical coding since no manipulations, mutilations or other stresses were applied. DNA extractions were performed as described in Zeisset & Beebee [40]. Four of the 17 populations were located in the glacial refugium area (REF, as per [12]) of Iberia and southern France, while the other 13 were located in the postglacial expansion area (PGE, as per [12]), all as inferred from mtDNA haplotype diversity [50].

**Table 1:** Summary statistics for MHC and microsatellite loci.

Populations	Microsatellites					MHC				
*Postglacial expansion (PGE) areas:*	*N*	*N_alleles_ (SD)*	*N_P_*	*R*	*H_E_*	*N*	*N_alleles_*	*N_P_*	*R*	*H_E_*
NW England (14)	40	2.1 (1.46)	0	2.0	0.30	29	2	0	2.0	0.49
SE England (15)	39	2.4 (0.98)	0	2.1	0.34	24	2	0	2.0	0.50
Ireland (13)	40	2.1 (1.35)	0	1.9	0.29	9	1	0	1.0	0.00
NW France (10)	40	2.0 (1.16)	0	1.9	0.26	12	2	0	1.8	0.08
NW France (11)	31	4.4 (1.62)	3	3.3	0.46	14	2	1	2.6	0.52
NW France (12)	39	3.4 (1.51)	0	3.2	0.47	22	3	1	2.9	0.53
Netherlands (16)	40	2.7 (1.70)	1	2.5	0.36	17	2	0	1.5	0.06
Germany (17)	40	3.6 (2.44)	3	3.1	0.46	27	3	0	3.0	0.57
Poland (18 & 19)	40	1.9 (0.69)	0	1.7	0.21	13	2	0	1.9	0.15
Estonia (22)	37	1.4 (0.79)	1	1.4	0.14	21	1	0	1.0	0.00
Denmark (20)	37	1.29 (0.49)	0	1.3	0.11	14	1	0	1.0	0.00
Sweden (21)	40	1.7 (0.95)	0	1.5	0.14	24	2	0	2.0	0.51
Switzerland (8 & 9)	40	2.86 (0.69)	1	2.7	0.50	30	5	3	3.5	0.60
**PGE means (SD)**	**38.7 (2.46)**	**2.5 (1.22)**	**0.69 (0.99)**	**2.2 (0.69)**	**0.31 (0.14)**	**19.7 (6.68)**	**2.2 (1.07)**	**0.38 (0.84)**	**2.0 (0.80)**	**0.31 (0.26)**
**PGE total numbers**	**503**	**64**	**9**	**–**	**–**	**256**	**12**	**5**	**–**	**–**
***Refugia (REF) areas***	***N***	***N_alleles_ (SD)***	***N_P_***	***R***	***H_E_***	***N***	***N_alleles_***	***N_P_***	***R***	***H_E_***
SW France (6)	16	5.3 (1.11)	5	4.6	0.67	13	5	2	5.6	0.81
SE Spain (4)	11	4.7 (1.60)	1	4.5	0.68	–	–	–	–	–
N Spain (5)	12	4.7 (1.11)	5	4.6	0.73	–	–	–	–	–
SW Spain (2)	36	9.3 (2.56)	12	6.7	0.79	10	4	0	4.0	0.78
SW Spain (3)	–	–	–	–	–	13	14	11	10.8	0.91
Portugal (1)	–	–	–	–	–	33	12	8	7.6	0.84
SE France (7)	22	5.9 (1.95)	3	4.9	0.65	–	–	–	–	–
**REF means (SD)**	**19.4 (9.16)**	**6.0 (1.67)**	**5.2 (3.65)**	**5.0 (0.93)**	**0.70 (0.06)**	**17.3 (9.18)**	**8.8 (4.99)**	**5.25 (4.43)**	**7 (2.93)**	**0.84 (0.06)**
**REF total numbers**	**97**	**99**	**26**	**–**	**–**	**69**	**30**	**21**	**–**	**–**

Summary statistics for MHC and microsatellite loci of *B. calamita* populations. The numbers in the population column refer to sampling sites in Figure 1. N is sample size and N alleles is mean number of alleles with standard deviations given for microsatellite loci, N_P_ is the number of population- specific alleles occurring only in one population, R is the allelic richness adjusted to the minimum sample size of nine individuals. H_E_ is mean expected heterozygosity.

### MHC Genotyping

MHC class II locus B β chain exon 2 sequences of *B. calamita* were amplified using primers located in the flanking intron regions. This is the only functional MHC class II locus thus far identified in *B. calamita* [42], [43]. The forward primer 2F347 (GTGACCCTCTGCTCTCCATT) with reverse primer 2R307b (ATAATTCAGTATATACAGGGTCTCACC) amplified a sequence of 279–282 base pairs (excluding primers). The 20 µl PCRs contained approximately 25 ng DNA, 0.4 µM of each primer, 100 µM dNTPs, 1x reaction buffer and 0.5 U of New England Biolabs *Taq* DNA polymerase. Thermal cycling consisted of an initial denaturation step of 94°C for 3 min and a touchdown protocol with a total of 35 cycles and ending with a elongation step of 72°C for 10 min. Each cycle started with a denaturation step of 94°C for 30 sec and ended with an elongation step of 72°C for 40 sec. Annealing temperatures consisted of 2 cycles at 62°C, 2 cycles at 60°C, 2 cycles at 58°C and 29 cycles at 56°C, each for 30 sec. Individual alleles were identified by SSCP analysis as described in Zeisset & Beebee [40]. Bands were excised from gels, re-amplified following Sunnucks *et al*. [52] and sent away for sequencing (Macrogen, Korea or Oxford Biochemistry Dept, UK). To reduce the risk of including PCR artefacts each allele was sequenced at least twice, either from different individuals or from two separate PCRs.

### Microsatellite Data

For comparative purposes we used microsatellite data obtained in previous studies from eight polymorphic loci in 600 individuals sampled from 18 populations [50], [51] and distributed over the entire biogeographical range (Figure 1, Table 1). To minimize PCR and scoring errors a small subset of samples with high incidences of non-amplification or difficult to score alleles were run twice. Microsatellite data were tested for the presence of null alleles and scoring errors using Micro-Checker 2.2.3 [53] and for effects of selection using the F_ST_ outlier approach implemented in LOSITAN [54], [55].

### MHC Sequence Analysis

To determine intron/exon boundaries we combined the putative intron and exon sequences (Genbank nos.: JX258913 and JX258914) to obtain a 532 base pair sequence of *B. calamita* locus B allele ‘Buca B2’ and used NNSPLICE version 0.9 [56], as implemented on http://www.fruitfly.org/seq_tools/splice.html, to predict splice sites.

Sequences were aligned and edited manually using Bioedit v. 7.0.9 [57]. The relative rates of non-synonymous (dN) and synonymous (dS) base pair substitutions were calculated according to Nei & Gojobori [58] applying the Jukes Cantor correction [59] for multiple hits in Mega 5 [60]. This was done for all sites and just for putative antigen binding sites (ABS), assuming functional congruence to human ABS identified by Tong *et al*. [61]. We used a Z-test [62] for positive selection. We also calculated average pairwise nucleotide distances (Kimura 2-parameter model, K2P) and Poisson-corrected amino acid distances [63] for ABS, non-ABS and all sites in Mega 5 with 1000 bootstrap replicates to calculate standard errors for the distance measures.

To identify specific sites under selection we used two new methods: a mixed effects model of evolution (MEME) to identify instances of both episodic and transient positive selection at individual sites [64] as well as a fast unbiased Bayesian approximation (FUBAR), both implemented on http://www.datamonkey.org [65]. MEME is superior at detecting sites where episodic positive selection is likely to be occurring [64]. For these analyses we used 282 bp (94 amino acids) of 57 locus B alleles of three species, *B.calamita*, *Bufo bufo* and *Bufo (Pseudoepidalea) viridis* (Genbank nos.: HQ388288, HQ388291, JX258874–JX258913, JX046488–JX046501, JX258919). After testing for recombination, a phylogenetic tree was inferred and used as the input for selection on particular codons using the two methods.

To investigate the evolutionary relationship between the MHC loci in the three *Bufo* species we used three methods were to test for signatures of recombination. This analysis was carried out for 282 bp of exon 2 sequence as well as for the 157 bp we used in phylogenetic tree reconstruction to investigate the effects recombination may have on tree construction. For this analysis we also included *B. calamita*, *B. bufo* and *B. viridis* locus A alleles (locus A is a putative non-functional locus, identified in an earlier study [43], (Genbank nos.: JX258916, JX283352, JX283353, JX258920, JX046502–JX046504) and used GENECONV [66] and MaxChi2 [67], both implemented in RDP3.44 [68]. Both of these methods performed well in a comparison of 14 recombination detection methods [69]. We applied Bonferroni correction for multiple tests and used automask sequences to optimize our dataset and to reduce the severity of the multiple testing correction. In addition we used a genetic algorithm recombination detection method (GARD; [70]), as implemented on http://www.datamonkey.org/GARD.

### MHC Phylogeny

We constructed a phylogenetic tree to visualize the relationship between anuran MHC class II β exon 2 alleles based on a total of 51 unique exon 2 sequences from 14 species: *Bufo bufo, B. viridis, Bombina bombina, B. variagata, B. pachypus, Alytes obstetricans, Xenopus laevis*, *Rana temporaria, R. catesbeiana, R. yavapaiensis, R. clamitans. R. sylvatica, Sachatamia ilex* and *Espadarana prosoblepon* [43], [39], [41], [2], [46], [44] in the NCBI database, in addition to a selection of our own from this study chosen to include the some of the most diverse sequences. Sequences were trimmed to 157 bp to match available data from the published exon 2 sequences. The urodele *Ambystoma tigrinum* and *Triturus cristatus* MHC sequences were included as outgroups. To investigate the evolutionary relationship among the 38 *B. calamita* locus B sequences from this study we constructed another phylogenetic tree, using 282 bp of sequence and *X. laevis* as an outgroup. For both trees evolutionary history was inferred using the Maximum Likelihood method based on the Kimura 2-parameter model [71] in Mega 5 [60]. Other tree building methods were also tested and provided congruent results (data not shown). A consensus tree was inferred from 1000 replicates [72]. As recombination and possible gene duplication can affect phylogenetic trees we also constructed a phylogenetic network using the program SplitsTree4, which can account for conflicting signals from recombination and gene duplication [73], [74] for the *B. calamita* MHC class II locus B. We used Jukes-Cantor distances and the Neighbor-Net method. For a network depicting the relationship between all three *Bufo* species’ MHC sequences see [43].

### Population Genetics

Compliance with Hardy-Weinberg equilibrium (HWE) in each population was assessed for microsatellite and MHC loci by applying the exact tests in Genepop 4.0.10 [75]. Linkage disequilibrium of the microsatellite markers was also tested in Genepop.

F-statistics [76], pairwise multilocus permutation tests of population differentiation, expected heterozygosity (H_E_) and allelic richness (i.e. the mean number of alleles corrected for samples size; R), were estimated for each population and overall in FSTAT 2.9.3.2 [77]. We used F_ST_ primarily as a way to measure the level of differentiation between populations. As F_ST_ may be affected by highly variable markers such as microsatellites we also calculated D_EST_ [78] in GenAlEx 6.5b3 [79], [80]. Pairwise comparisons of F_ST_ and D_EST_ within and between each class of loci, as well as isolation by distance (using ln distance vs. F_ST_/1−F_ST_), were made using the Mantel test facility in Genepop with 1000 permutations. Distances were measured using Google Earth between all sampling sites north of the Pyrenees (the region of postglacial expansion) that were not separated by sea, as most amphibians cannot survive seawater exposure. Distances were otherwise direct (Euclidean) allowing for bends to avoid sea water where necessary.

To investigate how variation was partitioned within and among REF and PGE populations we carried out an AMOVA in Arlequin v. 3.5 [81] for microsatellite and MHC data.

Phylogeographic relationships among the populations based on allele frequencies were determined separately for microsatellite and MHC data using Phylip v. 3.66 [82]. The analysis employed Cavalli-Sforza chord distances and the UPGMA algorithm with 1000 bootstraps for the multilocus microsatellite data.

Standard statistical tests for differences in allelic richness (R) and expected heterozygosity (H_E_) between REF and PGE populations as well as correlations between microsatellite and MHC data and microsatellite and allelic richness (R) and geographic distance were carried out using Statistix7 (Analytical Software, Tallahassee, USA).

## Results

### MHC Locus B Characteristics

**Figure 2:**
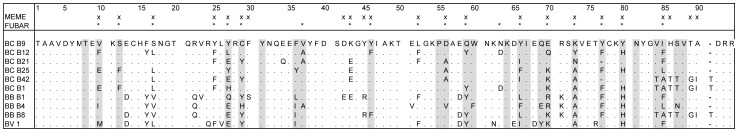
Amino acid alignment of a subset of MHC class II sequences. MHC class II amino acid sequences of *B. calamita* (BC), *B. bufo* (BB) and *B. viridis* (BV). Position 2 is the first amino acid position in exon 2 in these species according to splice site analysis. Position 5 corresponds to the first amino acid position of the second exon in the human MHC locus HLA-DRB. Shaded columns represent putative antigen binding sites (ABS) according to Brown *et al.* (1993) and/or Tong *et al.* (2006). Sign ‘−’ at position 77 denotes a codon deletion; signs ‘x’ and ‘*’ indicate amino acid positions under positive selection as determined by a mixed effects model of evolution (MEME) and a fast unbiased Bayesian approximation (FUBAR), respectively. The positive selection analysis was based on 57 alleles from locus B.

Splice site analysis for locus B predicted intron/exon boundaries between base pairs 3 and 4 after the 3′ end of the forward primer binding site and between base pairs 281 and 282 (2 bp into the reverse primer binding site), making the putative exon 279 base pairs long. These sites corresponded to exon 2 boundaries found in some other amphibian species [39]. There was evidence of historical positive natural selection at ABS sites in *B. calamita* (P = 0.002, Z = 2.973). Using MEME (P<0.1) we identified 23 positively selected codons in exon 2 and using FUBAR with a posterior probability >90% we found 19. Codons identified by both methods largely corresponded to putative antigen binding sites (ABS) as defined by Brown *et al*. [83] and Tong *et al*. [61] for the human MHC locus HLA-DRB (Figure 2). Average nucleotide distance over all nucleotide sequence pairs in exon 2 was 0.094 (SE 0.012) in *B. calamita.* Average amino acid distance at this locus was 0.149 (SE 0.028). Nucleotide and amino acid distances were much higher in the putative ABS (nucleotide: 0.376, SE 0.067; amino acid: 0.787, SE 0.159) than in non-ABS (nucleotide: 0.058, SE 0.010; amino acid: 0.083, SE 0.021) sites.

Of the 38 unique MHC class II exon 2 DNA sequences (Genbank nos.: HQ3882288, HQ388289, JX258875–JX258911) from locus B in 17 populations of *B. calamita*, ten showed evidence of a recombination event including a codon insertion towards the 3′ end resulting in the addition of threonine. Five had a deletion of three nucleotides which corresponded to a codon deletion in the MHC class II DAB alleles of the great crested newt, *Triturus cristatus* (Trcr-DAB*06, 08, 12, 15, 17. 19, 20 and 24, [84]) in Europe, as well as in the glass frog *Espadarna prosoblepon* (Espr-DRB*26, [44]) in Central America. In all three species phenylalanine or tyrosine was lost as well as a number of others in the case of the glass frog.

Recombination tests GeneConv and MaxChi detected between two and three recombination events among all the whole exon 2 MHC sequences, with between five and 15 recombination signals. GARD on the other hand detected no evidence of recombination. For the 157 bp sequences used in phylogenetic tree reconstruction only MaxChi detected one recombination event, with 13 recombination signals.

**Figure 3:**
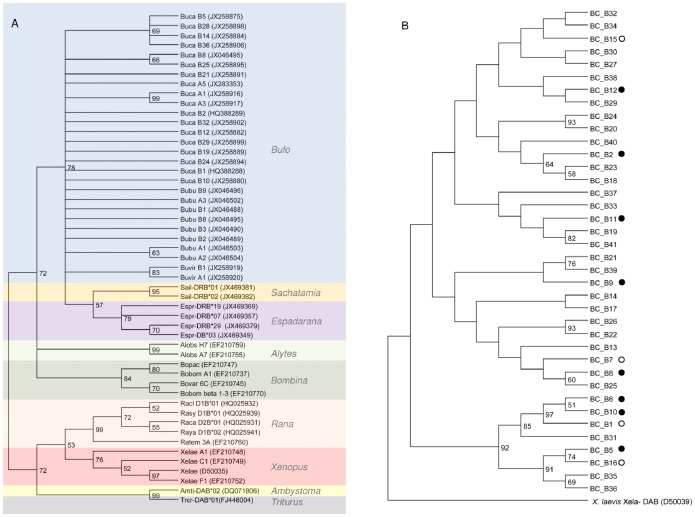
Phylogenetic tree of anuran exon 2 MHC class II nucleotide sequences. A: Multispecies comparisons using 157 bp of sequence. *Triturus* (Trcr) and *Ambystoma* (Amti) sequences were used as outgroups. Genbank accession numbers are given in brackets. B: *B. calamita* alleles with *Xenopus* outgroup using 282 bp of sequence. Filled circles, alleles only found in the PGE; open circles, alleles only found in SW France and PGE. Remaining alleles were only found in the REF populations. A ML bootstrap consensus tree from 1000 replicates [82] was constructed in Mega 5 [60]. The evolutionary distances were computed using the Kimura 2-parameter method [71]. Only bootstrap values above 50% are shown.

Amphibian MHC class II exon 2 sequences formed some strongly supported clusters (Figure 3A). *Rana*, *Xenopus* and Discoglossoidea (*Bombina* and *Alytes* species) sequences all formed separate groups. Within those groups there was also strong support for some branches separating species. The Central American *Sachatamia* and *Espadarana* clustered strongly with the European *Bufo*. Both the phylogenetic network (Figure S1) and the tree (Figure 3B) produced congruent results for the *B. calamita* MHC class II B locus.

### Population Genetics

For the MHC locus there were a total of 12 alleles in 256 individuals in the PGE group, but 30 alleles in 69 individuals in the REF group. There was a remarkably high number of population- specific alleles (25 out of 38, 66%) in the MHC data, each of which was found in a single population (Table 1). Twenty six alleles (68%) including 20 population- specific alleles (80%) were only found in the REF group and eight alleles (five population- specific) only in the PGE group. Only four alleles (10%) occurred in both groups, though in the REF group they were only found in the SW France population and not in any of the Iberian populations. None of the alleles found in Iberia occurred north of the Pyrenees and vice versa. However, the alleles in the REF and PGE groups did not cluster as similar sequences (Figure 3B) implying a common ancient ancestry for the alleles occurring in both groups combined. Three of the 17 populations (Germany, Spain (Doñana) and Portugal) were not in HWE at the MHC locus, in all cases due to a homozygote excess. As selection can generate divergence from HWE, we did not exclude these populations from further analysis. Although the presence of null alleles could not be ruled out, the fact that the primers were located in relatively conserved intron sequences means that null alleles are unlikely to be the cause of the deviation from HWE. Among the microsatellites LOSITAN identified one locus (Bcalµ8) as possibly affected by positive selection and we therefore excluded this marker from subsequent analysis. The number of alleles in the remaining seven microsatellite loci ranged from 8 (Bcalµ2) to 25 (Bcalµ3). Diversity measures for microsatellite and MHC loci are given in Table 1 and allele frequencies in Table A in File S1. Almost all of the microsatellite alleles were encountered at least twice, either in the same or in different populations and errors due to PCR or scoring are likely to be small. PCR repeats of individuals never gave conflicting results. Again, diversity was lower in the PGE group than in the REF group. There were 35 population- specific microsatellite alleles out of a total of 118 (30%) across all loci. A total of 52 alleles (44%, range 29–50%) including 26 population- specific alleles (74%) were found only in the REF group and 16 alleles (nine private) only in the PGE group. Across all loci fifty alleles (42%, range 25–50%) occurred in both groups. No microsatellite locus therefore showed allelic differentiation north and south of the Pyrenees as great as that shown by the MHC locus.

Twenty-four of 126 population × microsatellite loci comparisons showed significant deviations from HWE after Bonferroni correction. In all cases this was due to a homozygote excess and Micro-Checker indicated the possibility of null alleles in several populations at Bcalµ1, 2, 3, 4 and 6 with estimated frequencies ranging from 0.09 to 0.32 ([85], estimator 1; Table B in File S1). The number of loci with homozygote excess was particularly high in the German and Spanish (Seville) populations. Null alleles may therefore be a cause of the deviation from HWE. Another possible explanation is that in some populations a proportion of the samples consisted of siblings, although measures were taken to avoid sampling family groups [50]. However, as this was a small proportion of the total number of populations we did not exclude these from further analysis (see also [50]).

After Bonferroni corrections there were no cases of linkage disequilibrium among the loci.

### Comparison of MHC and Microsatellite Variation

**Figure 4:**
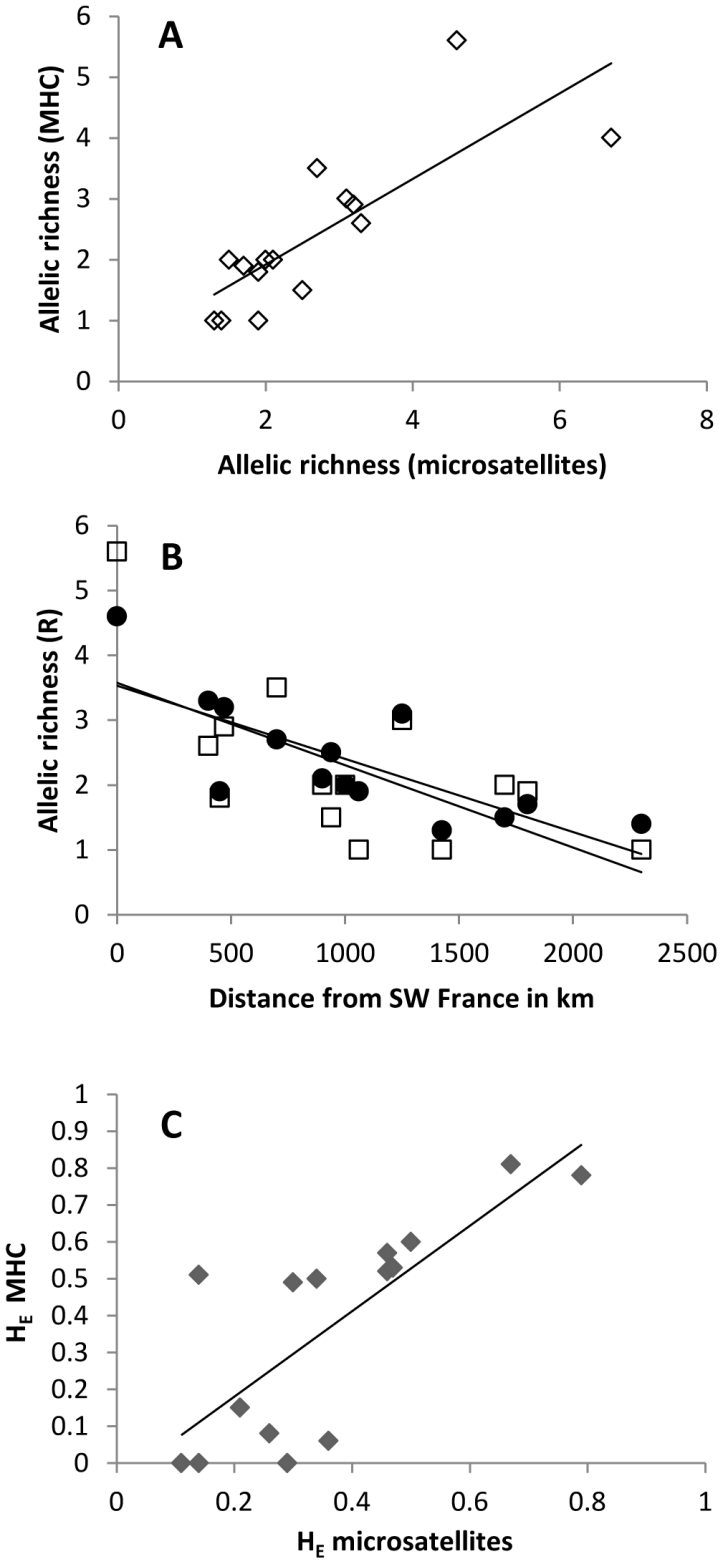
MHC and microsatellite diversity comparisons. A: Correlation of MHC and microsatellite allelic richness (R). B: Microsatellite (□) and MHC (•) allelic richness (R) and distance from SW France. C: Correlation of MHC and microsatellite expected heterozygosity (H_E_).

For both microsatellite and MHC markers most variation was accounted for at the within-population level (49% and 42% respectively) or among populations within REF and PGE groups (39% and 51% respectively, Table 2). However, the mean number of alleles corrected for sample size (allelic richness, R) was significantly higher in the REF than in the PGE for both microsatellite and MHC loci (MHC: χ^2^ = 4.20, DF = 1, P = 0.0404; microsatellites: χ^2^ = 6.92, DF = 1, P = 0.0085). MHC and microsatellite allelic richness was significantly correlated among populations where both types of loci were sampled (Figure 4A, r_s_ = 0.8371, n = 15, P = 0.0001). Microsatellite and MHC allelic richness outside Iberia declined with distance from the SW France refugium area (Figure 4B, r_s_ = −0.7899, n = 14, P = 0.0013). Expected heterozygosity between the types of loci was also significantly correlated (Figure 4C, r_s_ = 0.8312, n = 15, P = 0.0002). None of the correlations were unduly influenced by populations with a significant amount of null alleles (see Figure 4A and Figure 4C as well as Table A and Table B in File S1) and the correlations between heterozygosity and allelic richness for both types of loci were also significant excluding SW Spain and Germany from the analysis (H_E_: r_s_ = 0.7552, n = 13, P = 0.0001; R: r_s_ = 0.7872, n = 13, P = 0.000). Average F_IS_ values of MHC and microsatellite loci across all populations in this PGE zone were low in both cases (0.019 and 0.028 respectively) and not significantly different (Wilcoxon signed rank test, P = 0.603).

**Table 2:** AMOVA results for MHC and microsatellite loci.

	Among groups	Among populations within groups	Within populations
microsatellites	11.86*	39.44*	48.69*
MHC	6.37*	42.38*	51.25 ns

Percentage of variation explained by among group (PGE and REF), among population within groups and within population. Associated significance values of variance components based on 1023 permutations: ns = non significant, * = P<0.001.

**Figure 5:**
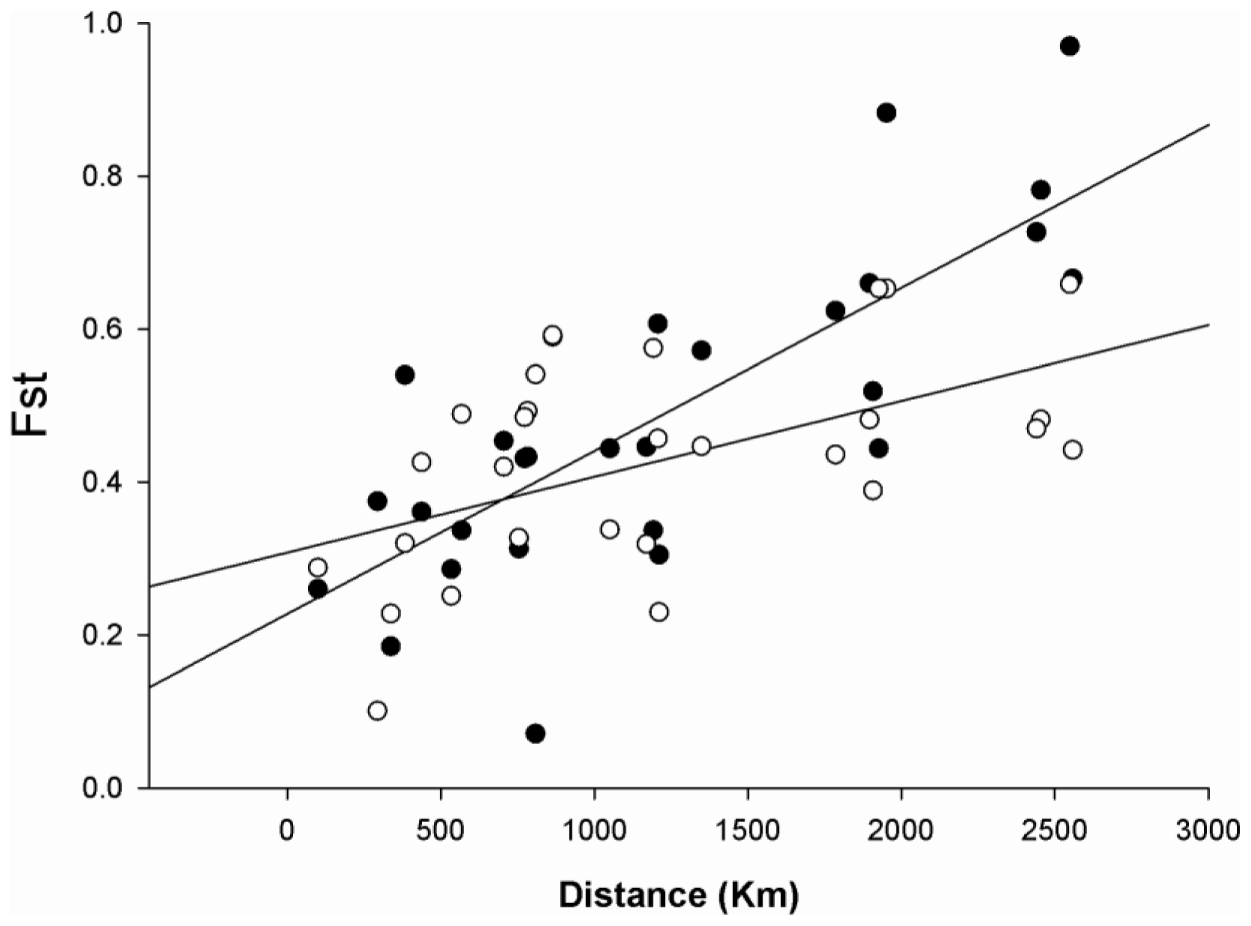
Relationships of pairwise F_ST_ and distance estimates. Microsatellite data are represented by open circles and MHC data by filled circles.

Pairwise F_ST_ comparisons among populations indicated significant population differentiation between 128 of the 136 population comparisons for MHC loci and between all but three for the microsatellite data (see Table S1). Using Mantel tests, pairwise F_ST_ and D_EST_ were significantly intercorrelated both for MHC and microsatellite loci for the PGE region including SW France (MHC: r_s_ = 0.6372, n = 105, P<0.0001; microsatellite: r_s_ = 0.2548, n = 105, P = 0.0089). However, in several cases where geographical separation was high, MHC D_EST_ = 1 thus providing incomplete resolution of differentiation level. Subsequent comparisons therefore focused on pairwise F_ST_ estimates which were correlated between MHC and microsatellite loci (r_s_ = 0.3128, n = 105, P = 0.0012). Excluding Iberian populations, among areas analysed for both microsatellites and MHC genotypes and not separated by seawater (n = 8; Bordeaux, SW France; Zurich, Switzerland; Carnac, France; Penmarch, France; Cherbourg, France; Halle, Germany; Bukowno/Bielowieza, Poland; Parnu, Estonia), the correlation between MHC and microsatellite pairwise F_ST_ estimates was also strong (r_s_ = 0.432, P = 0.025). Mean pairwise F_ST_ estimates in this region were similar for both types of loci (0.428 for microsatellites, 0.487 for MHC) and there was significant isolation by distance (IBD, P<0.0001 in both cases). However, the strength of IBD was greater for MHC than for microsatellites as shown using untransformed F_ST_ and distance estimates in Figure 5. Regression slopes for the two loci were significantly different (F = 6.14, P = 0.0165). This strongly indicates a role of selection in shaping MHC diversity, as the effects of drift on microsatellite F_ST_ estimates are expected to be greater than those on MHC loci, due to their higher mutation rates.

**Figure 6:**
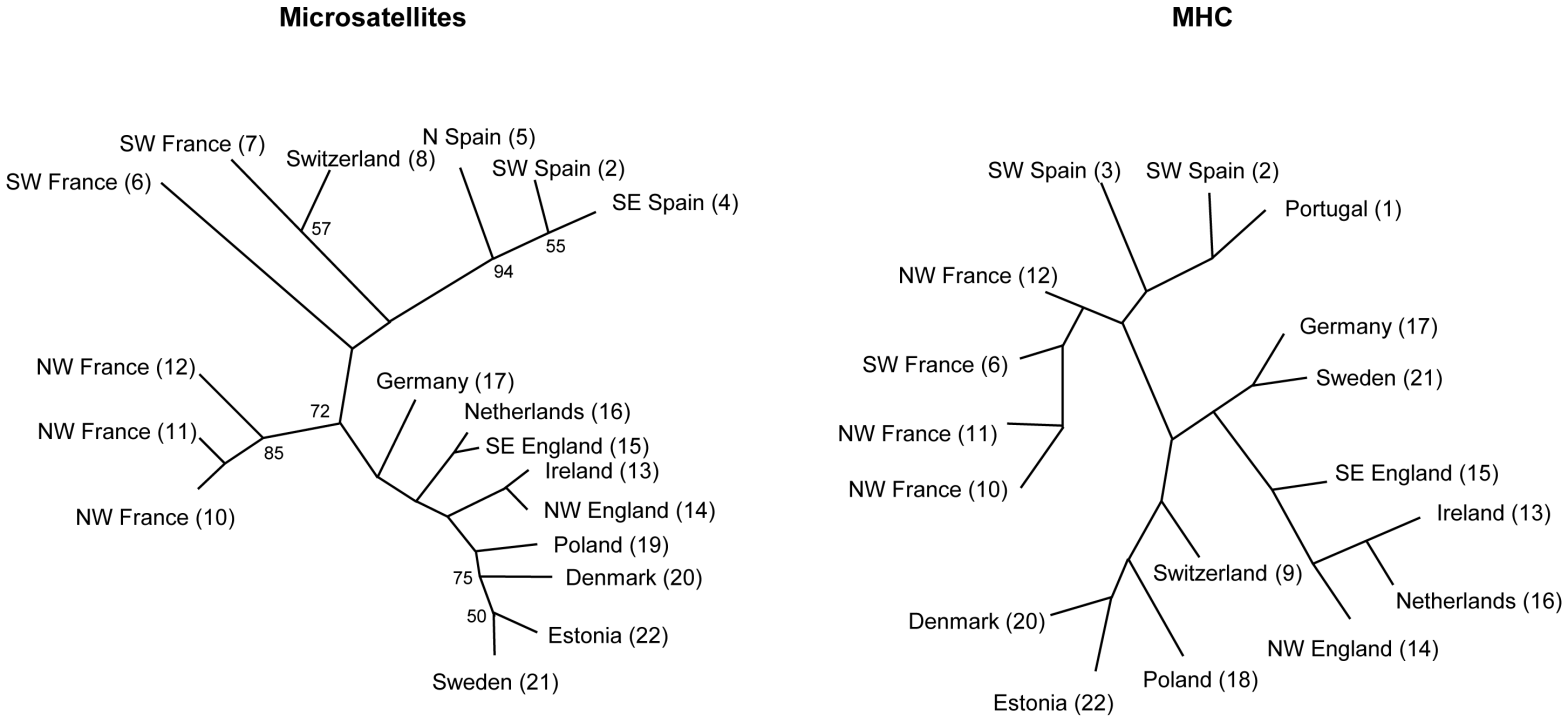
Phylogeography of *B. calamita* populations. Bootstrap values >50% are given for the microsatellite analysis. Sampling site numbers corresponding to Figure 1 are given in brackets.

**Figure 7:**
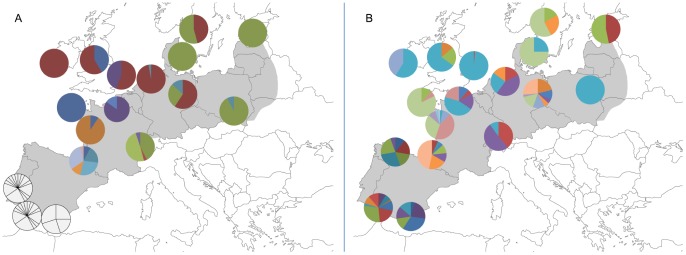
Allele frequency distributions in the PGE area. A: MHC locus allele frequency distributions. Colour coding for the Iberian populations is excluded from this figure due to the large number of population specific alleles present; B: Bcalµ3 locus allele frequency distributions. Maps modified from d-maps.com.

Phylogeographic trees based on microsatellite and MHC allele frequencies were broadly congruent (Figure 6). However, allele frequencies and distributions in the PGE region were significantly different between the loci (Figure 7). We excluded colour coding for the MHC locus in the Iberian populations from this comparison, as they do not share any alleles with the other populations and contain a large number of population specific alleles. For a full comparison see Table A in File S1 for allele frequencies at all loci in all populations. Certain MHC alleles were common in adjacent geographic areas (e.g Buca B2 in Ireland, UK, Netherlands, Germany and Sweden, Buca B5 in Sweden, Denmark, Estonia, Poland, Switzerland and Germany) (Figure 7A). No such pattern could be discerned for the most polymorphic microsatellite locus Bcalµ3 (Figure 7B).

## Discussion

Nucleotide distances in the MHC locus (0.094) were comparable to those found in other amphibian species, where diversity ranged from 0.062 in *Rana warszewitschii* to 0.155 *in R. catesbeiana* [41], [39], [40]. Many of the positively selected sites we identified in exon 2 corresponded to those involved in peptide binding in equivalent human MHC class II proteins [83], [61]. Many of these sites were congruent with human ABS identified by either Tong *et al*. [61] or Brown *et al*. [83] (Figure 2). The others were located outside the human ABS and some of the human ABSs were not identified as positively selected sites in the *Bufo* MHC class II loci. Similar findings have been reported by others (e.g. [86], [87], [84], [41]), indicating that species- specific selection pressures were acting on the MHC genes. Two methods for detecting recombination in the *Bufo* MHC sequences indicated its occurrence in these species. This may explain the adjacent intron 2 sequence similarity found at locus A and B within *B. calamita* [43]. Although our phylogenetic analysis was based on only a short DNA sequence, it does reflect the current view of the phylogenetic relationship of amphibians [88], [89], [90]. Interestingly the Central American glass frog *Espadarana prosoblepon,* which clustered with *Bufo* in our phylogenetic tree, also showed remarkable sequence similarity to *Bufo* MHC sequences in the first 200 bp of intron 2, which basically consists of a 100 bp repeat [43], [44]. Although we did not sequence intron 2 of *B. viridis* or *B. bufo* the fact that the intron- specific primers amplify MHC loci in these species indicates at least some conservation at introns across taxa [43]. Blast searches did not find these sequences elsewhere. Such conservation across widely separate taxa may indicate some functional significance of this sequence. For example, the first 130 bp of intron 2 sequences of New World ranid species (*Rana catesbeiana, R. clamitans, R. pipiens, R. sylvatica, R. yavapaiensis, R. warszweitschii, R. palustris;* [41]) were also remarkably conserved across species, though no repeat was detected. Sato *et al*. [91] analysed 114 intron 2 sequences of passerine bird species and found that most of them largely consisted of repeat sequences, with a 10 bp repeat being particularly common. In addition they found a 60–80 bp DNA segment in intron 2 that occurred in noncoding segments of MHC sequences in a number of other passerine bird species. The function or role of repetitive sequences or conserved elements in intron 2 is not yet clear but clearly requires further study.

Of particular interest within the *B. calamita* MHC were alleles (all in Iberian REF populations) that had a codon deletion at the same position as found in the great crested newt *Triturus cristatus* in Romanian REF populations [12] as well as in the glass frog *Espadarna prosoblepon* (Espr-DRB*26, [44]) in Central America. It may be that selection pressure in colder climates eliminated these alleles from populations in North European amphibians, or that they confer advantages in warmer climates. The loss of these alleles by drift as populations expanded north cannot be ruled out but seems a remarkable coincidence for two unrelated taxa.

There was a clear difference in the levels of both MHC and microsatellite diversity between the REF and the PGE populations of *B. calamita*. The lack of shared MHC alleles between the REF and PGE populations was surprising and it is possible more shared alleles may be found in other REF populations. Nevertheless, for comparison, Babik *et al.* [12] found that populations of the great crested newt (*Triturus cristatus*) in post glacial expansion (PGE) areas possessed a subset of alleles from the refugia (REF) populations, when they compared three PGE populations from across Europe to only four small REF populations from Romania. Our data support the theory that natterjack toads survived the Weichselian glacial maximum 20 000 years BP in at least one north European refuge, most likely in France, and colonized northern Europe from there [50]. In *B. calamita* and *T. cristatus* variation of microsatellite and MHC loci was high in the REF groups and much lower in the PGE groups. A decrease in allelic diversity from southern to northern Europe is well documented in neutral loci (e.g. [92]). The high similarity in diversity distribution (decreasing in the PGE area as a function of distance from the REF area) and in phylogeographic patterns between the two types of loci imply that drift rather than selection was the dominant influence on MHC allelic variation at the biogeographic range scale. Mean F_IS_ estimates were close to zero for both classes of loci, with no signal of heterozygote excess as an indicator of diversifying selection in the MHC locus. A recent meta-analysis of the roles of natural selection and genetic drift in shaping MHC variation concluded that selection combined with drift during population bottlenecks can result in loss of MHC polymorphism at even greater rates than neutral genetic diversity [93]. Other studies have shown that microsatellite and MHC diversity is lost in similar proportions over time, with balancing selection unable to mitigate genetic drift [94] and that MHC diversity declines more steeply than microsatellite diversity after a bottleneck [95]. However, inconsistencies remain and in some cases selection can maintain polymorphism at MHC loci during population bottlenecks (e.g. [96]).

When comparing two different marker systems such as MHC loci and microsatellites it is important to consider the potential differences in the ages of observed alleles due to the higher mutation rates of microsatellites and potential back mutations. Microsatellite mutation rates in *B. calamita* have been estimated at a relatively low 1×10^−5^ [50], whereas the mutation rate at the DRB1 locus in chimpanzees is estimated to be 1.31×10^−9^ per site per year [97]. Assuming a similar mutation rate for the MHC locus B in *B. calamita* this would give a mutation rate of 1.1×10^−6^ for 279 bp of exon 2 per generation (three years), not much different from that of the microsatellites. Microsatellite mutation rates increase with microsatellite length and contractions become more likely than expansions as length increases [98]. As natterjack microsatellite length was generally higher in the PGE than in the REF area [50] it is possible that some variation was masked by back mutations generating homoplasy. However, the natterjack microsatellites were relatively short (mostly around 10–20 repeats with a maximum of 29 for Bcalµ3) and significant homoplasy was considered unlikely.

Despite the likely dominance of drift effects on *B. calamita* MHC variation across the species’ range there were some interesting differences between MHC and microsatellite genotype distributions that may imply an additional, albeit minor role of selection in structuring the MHC locus at this large geographical scale. MHC alleles were more sharply differentiated between the REF and PGE regions than were the microsatellite alleles. Isolation by distance effects in those parts of the mainland Europe PGE area where gene flow remained possible after postglacial sea level rise was significantly stronger for the MHC than for the microsatellite loci. The commonest MHC alleles in the PGE group were, on average, at higher frequency and more geographically clustered than the commonest alleles in the microsatellite locus with the most comparable allelic diversity. This pattern difference might imply weak effects of selection reflected in patterns of common MHC allele abundance in specific regions, such as north-west France, north-central Europe and eastern Europe, perhaps in turn reflecting local differences in pathogen communities. Pathogens often exhibit a ‘latitudinal diversity gradient’, with high diversity at the equator decreasing towards the poles [99]. For example, temperature is an indirect selective mechanism maintaining MHC diversity in wild salmon [18]. Therefore it is possible that the higher MHC diversity in the southern populations of *B. calamita* is maintained by higher pathogen- mediated selection pressure, but further studies into actual difference of pathogen prevalence in the various regions are needed to test this hypothesis.

Overall, our evidence clearly implies a stronger influence of drift than selection on this *B. calamita* MHC locus at the biogeographical scale. This is essentially similar to the situation discovered with a comparable study of another widespread European amphibian, *Triturus cristatus* [12]. Comparison of MHC and neutral loci in four populations of Atlantic herring (*Clupea harengus*) also failed to detect any evidence of selection acting on the MHC locus, although in this study a MHC-embedded microsatellite locus was used and it is not clear to what extent this reflected variability in the exon [100]. Marsden *et al.* [24] also found that although microsatellite diversity and MHC diversity were correlated in African wild dog populations, indicating genetic drift to be a major influencing factor, there were signatures of selection at the MHC locus. The apparent weakness of selective effects may however be influenced by the scale of the study. In Atlantic salmon drift and migration were more important than selection on large geographical scales but at smaller geographical scales the influence of selection was detected at MHC loci [101]. Postglacial colonisation with associated bottlenecks can evidently leave strong signatures of genetic drift long after the event. In contrast to this, the same MHC locus in a *B. bufo* population translocated over 400 km within the UK adapted within three generations to an allele frequency distribution similar to that of neighbouring populations in the receptor area [102], presumably reflecting selection in favour of the new conditions. A recent phylogeographic study of the bank vole (*Myodes glareolus*) found no spatial genetic structure among populations at MHC loci, but clear differentiation reflecting their major glacial refugia at a mtDNA gene. This may indicate yet another situation in which spatiotemporal variations in selection pressures acting over large areas can mask historical signals of population origins [103]. Other studies have also indicated that the mode and strength of selection acting on MHC diversity varies in time and space [104]. Further studies at small geographical scales including experimental translocations may prove fruitful in the investigation of selection on adaptive variation such as that expected with MHC loci, whilst no doubt further investigations into the role of pathogens in shaping MHC diversity remain necessary in isolating the evolutionary forces shaping MHC diversity.

## Supporting Information

Figure S1 **Phylogenetic network of MHC class II beta exon 2 sequences.** Neighbor-Net tree based on Jukes-Cantor distances of 282 bp of sequence of MHC locus B from *B. calamita.*
(TIF)

Table S1 **Population differentiation as estimated by F_ST_ values.** Numbers in brackets refer to population numbers in Figure 1. Non-significant values are indicated in red italics. A: Population differentiation as estimated by F_ST_ values for MHC data. P-values obtained after: 2720 permutations. Indicative adjusted nominal level (5%) for multiple comparisons is: 0.000368. B: Population differentiation as estimated by F_ST_ values for microsatellite data, based on 7 loci. P-values obtained after: 3060 permutations. Indicative adjusted nominal level (5%) for multiple comparisons is: 0.000327.
(XLSX)

File S1 Table A (sheet 1): Allelic frequencies in each population for microsatellite and MHC loci. Numbers in brackets refer to population numbers in Figure 1. Table B (sheet two): Brookfield 1 estimates of null allele frequencies at microsatellite loci.
(XLSX)
