# Supplementary figures and images for: Drift Rather than Selection Dominates MHC Class II Allelic Diversity Patterns at the Biogeographical Range Scale in Natterjack Toads Bufo calamita
Source: PLoS One. 2014 Jun 17;9(6):e100176. doi: 10.1371/journal.pone.0100176 (PMC4061088; doi:10.1371/journal.pone.0100176)

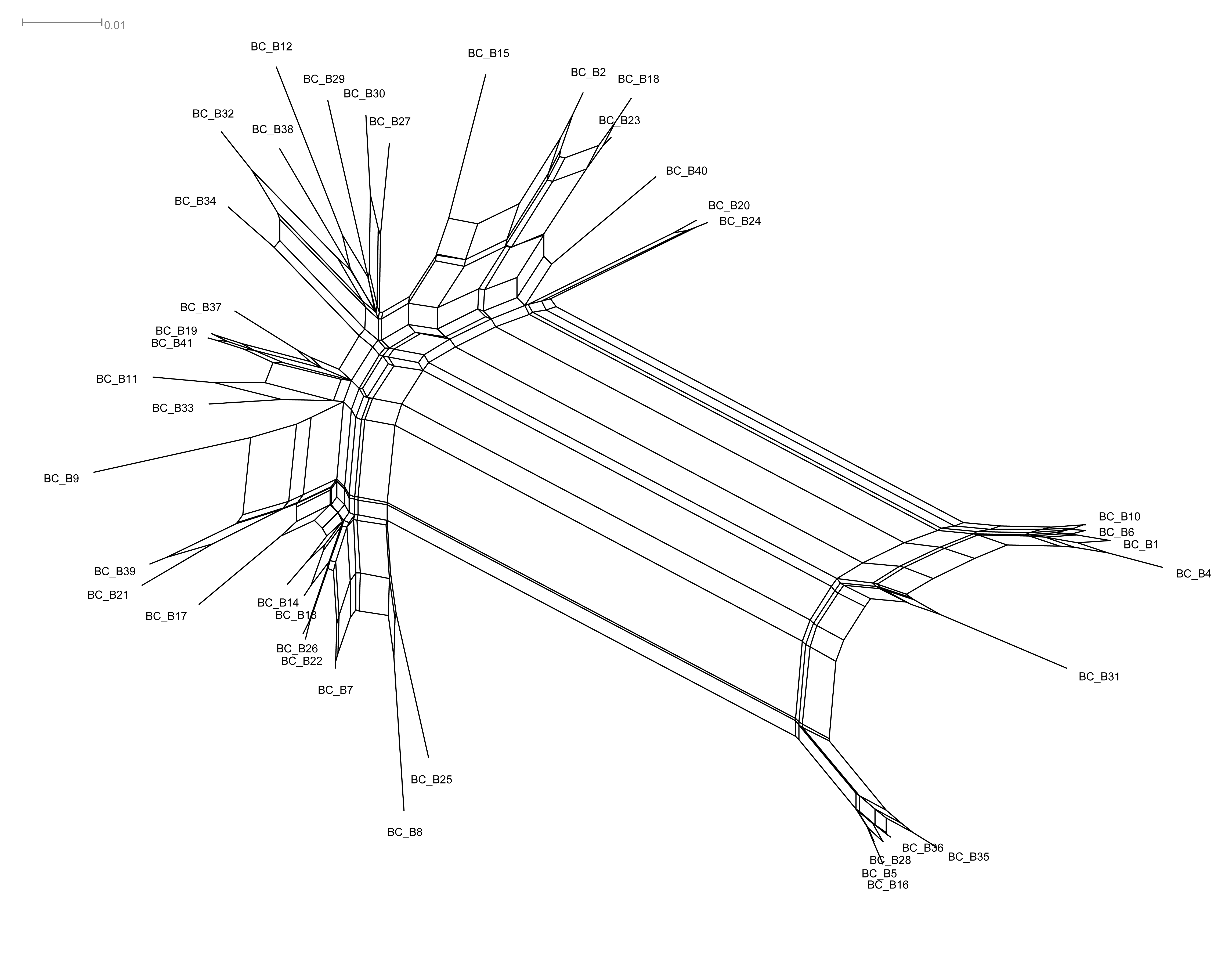

Supplement: Figure S1 — Phylogenetic network of MHC class II beta exon 2 sequences. Neighbor-Net tree based on Jukes-Cantor distances of 282 bp of sequence of MHC locus B from B. calamita. (TIF) [file pone.0100176.s001.tif]
